# Special Issue “Omics Research of Pathogenic Microorganisms”

**DOI:** 10.3390/genes14061229

**Published:** 2023-06-07

**Authors:** Peter van Baarlen

**Affiliations:** Host-Microbe Interactomics Group, Department of Animal Sciences, Wageningen University, De Elst 1, 6708 WD Wageningen, The Netherlands; peter.vanbaarlen@wur.nl

Infectious diseases of plants, animals and humans pose a serious threat to global health and seriously impact ecosystem stability and agriculture, including food security. In recent years, more serious damage by fungal diseases infecting plants and/or humans, and increased resistance of disease-associated fungi and bacteria to antimicrobial drugs have been reported. In this Special Issue (SI) “Omics Research of Pathogenic Microorganisms”, nine research groups have submitted a total of ten papers where they present and discuss results and insights on ecology, evolution and virulence of disease-associated microbes, host responses and an evaluation of two popular platforms to diagnose and monitor pathogenic microbes in complex microbiome samples. This SI highlights current -omics approaches and software tools that are widely used to study disease-associated microbes, from in vitro cultures to metagenomic samples and host–pathogen interactions. The main insights from the contributions to this SI are summarised below.

Lara and colleagues predicted metabolic interactions between bacteria associated with urinary tract infections (UTIs) and showed that a common dietary metabolite may influence population sizes of specific UTI-associated bacteria [1].

Interactions between bacterial clonal lineages were also one aspect of the study by Ruelens and de Visser, who showed that in vitro, in small populations of *Escherichia coli* bacteria, clonal interference and mutator phenotypes greatly influenced the rise of resistance to a β-lactam antibiotic [2] in these populations. Their study contributes to our understanding of evolution of antibiotic resistance in small “pioneer” populations of bacteria.

Next to antibiotic resistance, acid stress resistance is of considerable relevance to pathogenicity. Gu and colleagues used genomics to predict genes of interest to acid stress resistance of clinical *Salmonella* serotype Derby (S. Derby) and discovered 35 genes that may influence acid stress resistance in S. Derby, including CRISPR-Cas system genes. They validated, via in vitro bioassays, that two predicted operons including two CRISPR-Cas genes contributed to acid tolerance and virulence of S. Derby [3].

In addition to the abovementioned study by Gu and colleagues, bacterial CRISPR-Cas genes have been reported by diverse research groups to be associated with antibiotic resistance and virulence. Tanmoy and colleagues investigated genomes and metadata of clinical typhoid *Salmonella enterica* (S. Typhi) from Bangladesh for CRISPR-Cas sequence-derived biomarkers associated with antibiotic resistance of endemic isolates [4]. Tanmoy and colleagues reported candidate biomarker CRISPR-Cas genes, including one specific for extensively drug-resistant S. Typhi from Pakistan and Bangladesh, and discussed the possibilities and challenges of CRISPR-Cas biomarkers for the antibiotic resistance of endemic S. Typhi. Their findings were re-analysed and further discussed by Fabre and colleagues [5] and compared to their own previous work, emphasising the potential relevance of CRISPR-Cas typing for bacterial epidemiology. Tanmoy and colleagues addressed the discussion topics raised by Fabre and colleagues in a reply that is part of this SI [6].

Bacterial epidemiology, especially the sequencing of taxonomically informative ribosomal 16S rRNA gene sequences, greatly benefits from reliable sequencing platforms that can be used in-house, to facilitate the rapid and reliable typing of bacteria present in metagenomic samples. Heikema and colleagues compared Illumina short-read sequencing- and nanopore long-read sequencing platforms for 16S profiling. The authors used Oxford Nanopore sequencing technology (ONT) which has gained popularity for in-house sequencing at various labs. Heikema and colleagues came across potential pitfalls in standard ONT analysis pipelines and recommended validations to further improve reliable applications of long-read sequencing via ONT platforms [7].

Long-read sequencing was employed by Vilanova and colleagues to obtain high-quality genomes of *Monilinia* brown rot fungi that cause economically relevant diseases in stone fruits. They improved the resulting gene models using proteome and transcriptome data and listed 134 genes predicted to encode candidate virulence factors. Vilanova and colleagues assayed five predicted virulence factors and showed that three of these induced necrosis in plant leaf assays, neatly validating their -omics approach to predict fungal virulence factors [8].

Two other studies in this SI used transcriptome analysis to study pathogenic microbes and host responses to clinical bacteria. Byadgi and colleagues used transcriptomics of protozoan *Amyloodinium* parasites infecting the economically relevant fish seabass, to identify candidate virulence factors. Using their data as input in a virulence database, they focused on seven candidate virulence factors of which two were analysed in greater detail. Their transcriptome data provided a reference database for further study of *Amyloodinium* parasite biology [9].

Finally, Saha and colleagues used transcriptomics to unravel in vitro changes in gene expression of epithelial cells during the first 4 h of infection by highly virulent, clinical *Campylobacter jejuni* bacteria and an isogenic *cas9* gene deletion mutant. The authors identify several DNA damage, inflammatory and cell death pathways that were induced during infection by wild-type *C. jejuni*, but not during infection by the *cas9* gene deletion mutant, corroborating previous work that *C. jejuni* Cas9 has a role in the virulence of highly virulent *C. jejuni* strains [10].

This SI touches upon the diverse, highly relevant applications of -omics technologies to the study of pathogenic microbes and host–pathogen interactions. The editor hopes that the reports of this SI may further inspire scientists who are considering including -omics approaches in their research.
